# Rectal atresia with rectolabial fistula

**DOI:** 10.4103/0971-9261.43027

**Published:** 2008

**Authors:** S. P. Sharma, V. D. Upadhyaya, A. Pandey, A. N. Gangopadhyay

**Affiliations:** Department of Pediatric Surgery, Institute of Medical Sciences, Banaras Hindu University, Varanasi, India

**Keywords:** Rectal atresia, rectolabial fistula

## Abstract

Rectal atresia is a rare condition in which the anus and sphincter muscles are normally developed, with usually no fistulous communication with the urinary tract. We describe an unusual case of membranous rectal atresia with recto-labial fistula. It was treated by blind perforation of rectal membrane with lay opening of fistlous tract.

## INTRODUCTION

Rectal atresia is an extremely unusual type of anorectal malformation that is associated with a normal anal canal and usually no fistulous communication exists with the urinary tract.[1] The reported incidence is 1-2% of all anorectal anomalies. Most authors believe it to be an acquired lesion with a vascular genesis. One of the arguments quoted is the lack of other congenital anomalies. Several operative procedures are recommended for this lesion. We are reporting a very rare case of membranous rectal atresia with rectolabial fistula which was treated successfully.

## CASE HISTORY

**Figure 1:**
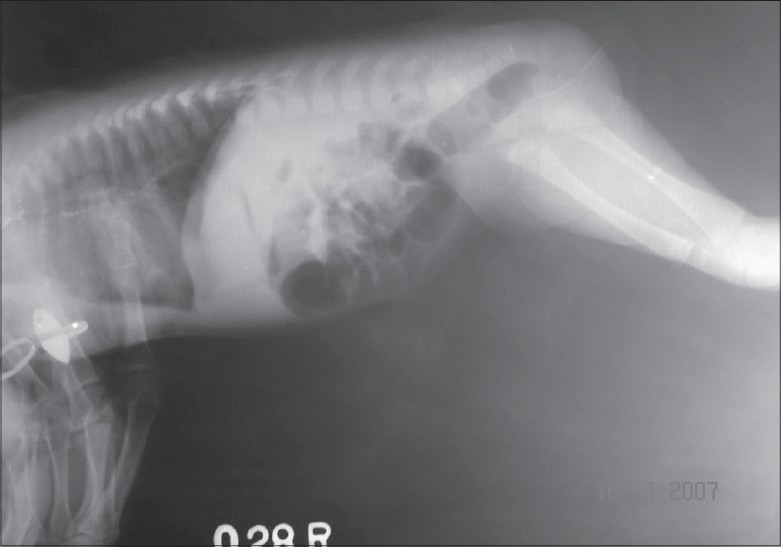
Invertogram

**Figure 2:**
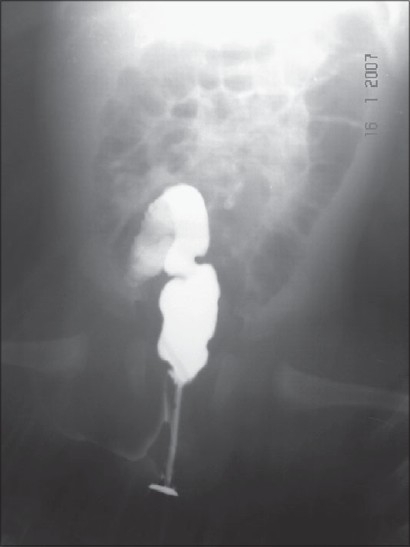
Fistulogram

A three-day-old female child was admitted with a history of not passing meconium since birth. On examination, abdomen was distended. Careful examination revealed normally placed anal opening, with normal fourchette and small fistulous opening in the lower third of left labia majora. There was no meconium or any type of discharge from the opening. The anal opening was calibrated with number 8 infant feeding tube and a resistance was observed at about 2 cm from the anal opening. X-Ray (invertogram along with feeding tube *in situ*) was suggestive of membranous rectal atresia [Figure 1]. The membrane was perforated blindly by using Hegar's dilator following which meconium was coming from the fistulous opening. Fistulogram revealed fistula between upper anal canal and the labia [Figure 2]. The fistulous opening was laid open under general anesthesia as it was a low level fistula. Patient was allowed orally after 48 h. She was discharged on third postoperative day in a satisfactory condition with proper follow-up advice. In follow-up there was no discharge from the operation site. Cosmetic appearance was satisfactory.

## DISCUSSION

Rectal atresia (RA) with a normal anus is a rare anomaly mostly described as part of a series of anorectal malformations[2] with a reported incidence of 1-2% [3] of all anorectal anomalies. In rectal atresia, the anus is open, but a variable segment of rectum superior to the anus is atretic and no fistula is present[1] or in other terms the terminal bowel (rectum) ends blindly at any level. The anus and anal canal are normal in correct relationship to the intrinsic sphincter and puborectalis.[4] We are presenting a case of membranous type rectal atresia (type 1) with fistulous communication at the labia which to the best of our knowledge is the first to be reported. In review of literature few cases of rectal atresia with rectal stenosis[5] and rectal atresia with recto-bulbar[6] urethral fistula had been reported. In our case fistulous opening was present at labia majora communicating with the rectum just distal to membranous atresia. The curative surgical management done in our case was lay opening of the tract and healing by secondary intention.

## CONCLUSION

Rectal atresia associated with fistulous communication is a very rare entity. However one should always look for any fistulous opening in the perineum and fistulogram can delineate between a high or low fistula. Management of low lying fistula is laying it open and that of high fistula is by standard posterosagittal anorectoplasty.
